# Microevolutionary Events Involving Narrow Host Plasmids Influences Local Fixation of Vancomycin-Resistance in *Enterococcus* Populations

**DOI:** 10.1371/journal.pone.0060589

**Published:** 2013-03-29

**Authors:** Ana R. Freitas, Carla Novais, Ana P. Tedim, María Victoria Francia, Fernando Baquero, Luísa Peixe, Teresa M. Coque

**Affiliations:** 1 REQUIMTE, Laboratório de Microbiologia, Faculdade de Farmácia, Universidade do Porto, Porto, Portugal; 2 Instituto Ramón y Cajal de Investigación Sanitaria (IRYCIS), Madrid, Spain; 3 CIBER en Epidemiología y Salud Pública (CIBERESP), Madrid, Spain; 4 Unidad de Resistencia a Antibióticos y Virulencia Bacteriana asociada al Consejo Superior de Investigaciones Científicas (CSIC), Madrid, Spain; 5 Servicio de Microbiologia, Hospital Universitario Marqués de Valdecilla e Instituto de Formación e Investigación Marqués de Valdecilla (IFIMAV), Santander, Spain; Universitätsklinikum Hamburg-Eppendorf, Germany

## Abstract

Vancomycin-resistance in enterococci (VRE) is associated with isolates within ST18, ST17, ST78 *Enterococcus faecium* (Efm) and ST6 *Enterococcus faecalis* (Efs) human adapted lineages. Despite of its global spread, vancomycin resistance rates in enterococcal populations greatly vary temporally and geographically. Portugal is one of the European countries where Tn*1546* (*vanA*) is consistently found in a variety of environments. A comprehensive multi-hierarchical analysis of VRE isolates (75 Efm and 29 Efs) from Portuguese hospitals and aquatic surroundings (1996–2008) was performed to clarify the local dynamics of VRE. Clonal relatedness was established by PFGE and MLST while plasmid characterization comprised the analysis of known relaxases, rep initiator proteins and toxin-antitoxin systems (TA) by PCR-based typing schemes, RFLP comparison, hybridization and sequencing. Tn*1546* variants were characterized by PCR overlapping/sequencing. Intra- and inter-hospital dissemination of Efm ST18, ST132 and ST280 and Efs ST6 clones, carrying rolling-circle (pEFNP1/pRI1) and theta-replicating (pCIZ2-like, Inc18, pHTβ-like, two pRUM-variants, pLG1-like, and pheromone-responsive) plasmids was documented. Tn*1546* variants, mostly containing IS*Ef1* or IS*1216*, were located on plasmids (30–150 kb) with a high degree of mosaicism and heterogeneous RFLP patterns that seem to have resulted from the interplay between broad host Inc18 plasmids (pIP501, pRE25, pEF1), and narrow host RepA_N plasmids (pRUM, pAD1-like). TAs of Inc18 (ω-ε-ζ) and pRUM (Axe-Txe) plasmids were infrequently detected. Some plasmid chimeras were persistently recovered over years from different clonal lineages. This work represents the first multi-hierarchical analysis of VRE, revealing a frequent recombinatorial diversification of a limited number of interacting clonal backgrounds, plasmids and transposons at local scale. These interactions provide a continuous process of parapatric clonalization driving a full exploration of the local adaptive landscape, which might assure long-term maintenance of resistant clones and eventually fixation of Tn*1546* in particular geographic areas.

## Introduction

Since its first description in the late 80’s, vancomycin-resistant enterococci (VRE) have been increasingly reported worldwide, but presenting remarkable geographical and temporal differences in local rates (http://www.cddep.org/ResistanceMap/bug-drug/EFa-VC) [1]-[3]. Vancomycin-resistant *Enterococcus faecium* (VREfm) became endemic in most North American hospitals since the mid 90′s [1], [2], [4]–[6] while their overall occurrence in Europe remained low until recently, when VRE nosocomial outbreaks started to be increasingly reported in some European countries (Annual Report of the European Antimicrobial Resistance Surveillance Network, EARS-Net, 2009) [1], [3], [7], [8]. Despite *E. faecium* (Efm) being less frequently found than *Enterococcus faecalis* (Efs) in clinical isolates, it is far more frequently resistant to vancomycin, one of the last-line intravenous antibiotic resources for therapy. However, although the rate of vancomycin-resistant *E. faecalis* (VREfs) has remained low, they are steadily increasing in both the US and in EU countries (http://www.cddep.org/ResistanceMap/bug-drug/EFe-VC) [3].

Vancomycin resistance among enterococci is mostly due to the spread of Tn*1546* (*vanA* genotype) and Tn*1549* (*vanB* genotype), which are generally identified on plasmids and chromosome, respectively [3]. The few studies in which plasmids carrying Tn*1546* from human or animal isolates were characterized revealed they belong to plasmid families RepA_N (pheromone-responsive plasmids and derivatives of pRUM and pLG1), Inc18 and pHTβ [9]–[18] suggesting an apparent plasmid promiscuity of this transposon influencing its dissemination among enterococcal populations.

Recent analysis of enterococcal populations in the clinical setting depicts a rugged epidemiological profile, with successive waves of isolates causing infections, which belong to specific lineages of *E. faecium* (ST17, ST18 and ST78, previously considered within the same clonal complex (CC) 17), and *E. faecalis* (ST6, ST40) [19]–[21]. However, regional differences in the rates of VRE cannot be only explained by clonal replacement dynamics as suggested for other pathogens [22]–[24].

The aim of this study was to address the dynamics of vancomycin resistance among enterococci in Portugal, one of the developed countries with higher rates of both VREfm (21–23%) and VREfs (1.8–4.1%) (www.earss.rivm.nl; http://www.cddep.org/ResistanceMap/bug-drug/EFe-VC), and where VanA is prevalent over VanB [3], [25]–[27], by analyzing the clonal and plasmid backgrounds influencing the spread and persistence of Tn*1546*. Our study suggests that clonalization, the local selection of distinct clonal variants giving rise to durable bacterial lineages, might result and be modified by the local spread and recombinatorial dynamics of mobile genetic elements, thus providing new clues about the local multi-hierarchical evolutionary biology of vancomycin resistance.

## Results

### Local dynamic landscape drives the spread and fixation of vancomycin resistance in Portuguese hospitals

**Figure 1:**
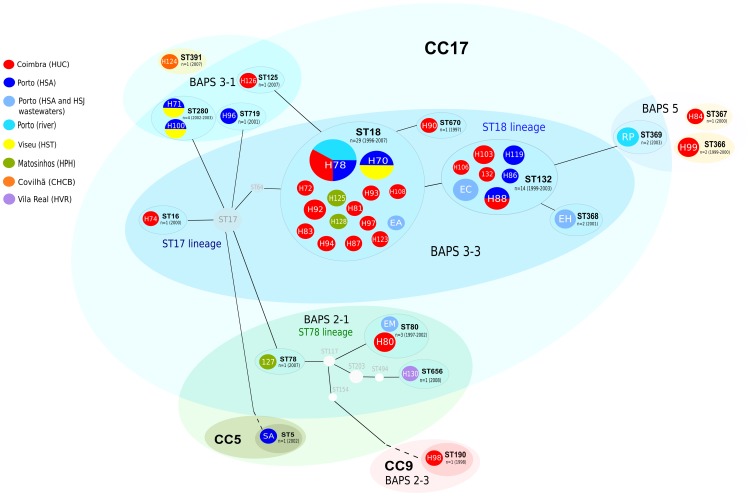
Population of vancomycin resistant *Enterococcus faecium.* Abbreviations: ST, sequence types; CC, clonal complex; BAPS, Bayesian Analysis of Population Structure; HUC, Hospital Universitário de Coimbra; HSA, Hospital Santo António; HSJ, Hospital São João; HST, Hospital São Teotónio; HPH, Hospital Pedro Hispano; CHCB, Centro Hospitalar da Cova da Beira; HVR, Hospital S. Pedro. A colored circle represents each PFGE type (white numbers/letters; H for hospital, SW for sewage, R for river and S for swine clones) and each PFGE type is associated with the corresponding sequence type (STs are represented in black letter and in colored elipses grouping different PFGE types) and BAPS group (in colored elipses grouping different STs). The size of the colored circles corresponds to the number of isolates. CC17 (in light blue), CC5 (in light green), CC9 (in light red) and the singletons ST366, ST367 and ST391 (light yellow) are represented according to the eBURST algorithm (download on 26^th^ January 2012) with black lines joining single locus variants (SLV). STs that were not identified in this study are represented as light grey nodes to link the sequence types identified in this study accordingly to eBURST. ST18 strains (H70, H78, H87, H93, H108, H125) and most ST132 strains (H86, H88, H106, SWC) were clonally related by PFGE (< 7 bands difference). Remarkable relationships among PFGE banding patterns of strains belonging to different STs were observed (H125/ST18 and H126/ST125; H124/ST391 and H71/ST280, SWM/ST80 and H86/H88/H106/H119/SWC/ST132, and isolates SWA/ST18 and SWC/ST132 (< 8 bands difference). This figure drawn up was performed in the “Open Source vector graphics editor Inkscape” (version Inkscape-0.48.2–1).

**Figure 2:**
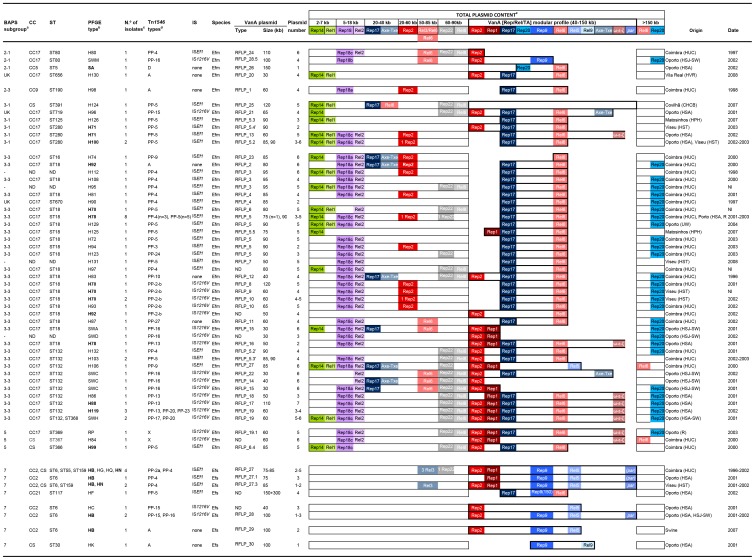
Clonal and plasmid diversity among VREfm and VREFs from Portugal. Abbreviations: IS, insertion sequence; Efm, *Enterococcus faecium*; Efs, *Enterococcus faecalis*; kb, kilobases; BAPS, Bayesian Analysis of Population Structure; ST, sequence type; CC, clonal complex; rep (replicases); rel (relaxases); TA (toxin-antitoxin system); HUC, Hospital Universitário de Coimbra; HSA, Hospital Santo António; HSJ, Hospital São João; HST, Hospital São Teotónio; HPH, Hospital Pedro Hispano; CHCB, Centro Hospitalar da Cova da Beira; HVR, Hospital S. Pedro; SW, sewage wastewaters; UW, urban wastewaters; R, river; ND, not determined; NI, not identified; UK, unknown. ^a^The distribution of the different isolates is shown by BAPS subgroups as described [19]. ^b^PFGE types shown in bold represented widespread clones in Portuguese hospitals and/or aquatic surroundings over years. ^c^Most Efm isolates expressed resistance to vancomycin, teicoplanin, erythromycin, ampicillin, ciprofloxacin (92–100%) and to a lesser extent to high levels of kanamycin (65%), gentamicin (41%), streptomycin and tetracycline (28% each). While *acm* was identified in different CC17 and non-CC17 lineages (76%), *esp* was detected in CC17 isolates (35%, ST132 and its SLVs ST368, ST369) and *hyl* was sporadically found (9%, ST18, ST125, ST132, SLVs of each other, and ST280 isolates) [25]. Efs isolates (mostly ST6) showed resistance to vancomycin, teicoplanin, erythromycin, ciprofloxacin, high levels of gentamicin and kanamycin (82–100%), tetracycline and chloramphenicol (65% each) and high levels of streptomycin (46%), and mostly contained *gelE* and *agg* (>90%), *cyl* (82%) and *esp* (46%) [26]. ^d^Tn*1546* designation is based on the results obtained by a PCR assay described by Woodford *et al.* consisting on the amplification of overlapped fragments covering the whole Tn*1546* [68]. Fragments of unexpected length were further analysed by sequencing (this study) [27]. ^e^The total rep/rel/TA content of isolates is represented according to its location on plasmids of different size ranges. Rep (normal cells), rel (cells with dots) and TA (cells with diagonal stripes) genes belonging to the same plasmid are represented with the same color and that belonging to the same plasmid family with the same range of colors. The content of VanA plasmids including rep, rel, and TA genes is indicated according to the plasmid type in which they were identified, as well as by the numeric nomenclature used by Jensen *et al*. [72] for replicases (rep_1_, rep_2_, rep_9_, rep_14_, rep_17_, rep_18a_), given new and consistent designations to replicases non described in reference 72 (rep_18b_, rep_18c_, rep_20,_ rep_22_). Relaxases were designated per numerical order as designed by M. V. Francia (unpublished data). Rolling-Circle plasmids are represented in green (rep_14/pRI1-like_, rel_1/pRI1_), small-theta replicating plasmids in violet (rep_18a/pEF418_, rep_18b/pB82_, rep_18c/pCIZ2_, rel_2/pCIZ2_), Inc18-like plasmids in different red tones (rep_1/pIP501_, rep_2/pRE25/pEF1_, rel_6/pEF1_, TA_Inc18-ω-ε-ζ_), RepA_N plasmids in different blue tones, pRUM in dark blue (rep_17/pRUM_, rel_3/pRUM_, TA_pRUM-Axe-Txe_), pLG1 in turquoise (rep_20/pLG1_), pheromone-responsive plasmids in light blue (rep_9/pAD1_, rel_5/pAD1_, rel_9/pCF10_, *par*_pAD1_), and pHTβ/pMG1 plasmids in grey (rep_22/pHTβ_, rel_8//pHTβ_). Rep families are named Rep_ ˝n˝_ where ˝n˝ indicates the number assigned to different rep-families according to Jensen *et al*. [72]. The name of the most representative plasmid of the family is also represented for a better follow-up of the results (e.g. rep_17/pRUM_, rep17 from pRUM and related plasmids p5753cB and pS177; rep_1/pIP501_ rep1 linked to Inc18 plasmids as pIP501, pIP816 and pRE25; rep_9/pAD1_, rep9 linked to pCF10, pAD1, pTEF1, pTEF2, pBEE99, pMG2200; rep_14/pRI1-like_, rep14 associated with RCR plasmids pEFNP1, pJS42 and/or pRI1; rep_18a/pEF418_, rep18 from pEF418; and rep_22/pHTβ_, rep of both pHTβ and pMG1 plasmids). We further specified the name of different plasmids associated with a given group if necessary. For example, it results helpful for Inc18 family given the number of plasmids containing the same rep gene. These plasmids are increasingly identified among isolates of different origins (e.g. rep_2/pRE25/pEF1_ for designing rep2, as rep and rel modules of pEF1, a plasmid originally identified in olives [35], seems to be widely present in all Efm clinical isolates)_._ Sequencing identified the different variants within these families (see text). Rep_18b_, rep_18c_ and rep_20_ were not included in Jensen's scheme [72] and the numbers were assigned in this paper following that numeration (rep_18b/pB82_, rep from pB82; rep_18c/pCIZ2_, rep from pCIZ2; rep_20/pLG1_, rep from pLG1). Rel genes were arbitrarily designated with numbers corresponding to different plasmid types [9] (Francia *et al*, unpublished data): Rel_1_, pJS42, pRI1; Rel_2_, rel from p200B, pCIZ2 and/or pB82 plasmids; Rel_3_, pRUM; Rel_5_, rel from pAD1, pTEF1, pAM373 and the pathogenicity island of V583; Rel_6_, pEF1; Rel_8_, pHTβ and pMG1; Rel_9_, pCF10. Toxin-antitoxin systems included Axe-Txe from pRUM, ω-ε-ζ from Inc18 plasmids and *par* from pAD1. Genes hybridizing in the same band as *vanA* plasmids appear in bold rectangles.

We have determined that the enterococcal population from the Portuguese hospitals is formed by an ensemble of MLST/PFGE clones. Efm isolates fit in three out of six phylogenomic groups recently established by using Bayesian Analysis of Population Structure (BAPS), namely BAPS groups 2, 3 and 5 [19] (Figure 1). Most of the isolates cluster into the predominant BAPS group 3 [subgroup 3–3 comprising main human lineages ST18 (ST18 and ST132) and ST17 (ST16); and subgroup 3–1 comprising ST280], and the BAPS group 2 (including ST80 and ST656/ST78 lineage, ST5/CC5, ST190/CC9), which have been previously associated with isolates from humans and both animals and humans, respectively [10], [19], [25], [28]–[30]. A number of clones cluster in the small Efm BAPS group 5 (ST366, ST367, ST369), which seems to comprise mosaic genomes [19]. Isolates of Efs belong to ST6/CC2, ST30, ST55, ST117, and ST159 lineages although, to the date of this publication, Efs population has not been clustered in different BAPS groups. Among all them, isolates within ST18 Efm and ST6 Efs lineages were predominant, in line with the intra- and interhospital spread of particular highly transmissible Efm and Efs clones recovered in Portuguese hospitals since the late 90s [22], [25], [31], [32]. While ST6 Efs was widely disseminated in all hospitals analyzed in this country [26], specific Efm lineages were overrepresented in Coimbra (ST18) and Oporto (ST132, a single locus variant, SLV, of ST18). Strains belonging to ST18 (showing PFGE types H70 and H78), ST132 (PFGE type H88) and ST280 (with PFGE types 71 and H100) were spread in different hospitals (Figure 1 and Figure 2).

It is worthwhile to note the possible relatedness between isolates of different STs (Figure 1 and 2). They include some isolates linked to BAPS 3–3 subgroup as ST18, ST80, ST125, ST132, ST368, ST369, all SLVs of each other, with PFGE patterns differing in less than 8 bands difference. Similarly, strains identified as ST280 and ST391, both linked to BAPS group 3–1, showed related PFGE patterns despite being trilocus variants (≤ 8 bands difference).

### *vanA*-Tn*1546* is located on highly transferable mosaic plasmids involving narrow host pRUM and pAD1 derivatives

The plasmid content of the isolates studied appears in Figure 2. Efm isolates carried a variable number of plasmids (n = 1–6) which contained specific sequences of different families including rolling-circle plasmids (RCR) related to pRI1 and small theta plasmids related to pCIZ2, RepA_N (pRUM-like, pLG1), pHTβ (present in all ST132 isolates), and Inc18 (pRE25 and pEF1-related). All Efs contained RCR plasmids and pheromone responsive-plasmids.

*vanA*-Tn*1546* was located on plasmids ranging from 30 to 150 kb, successfully transferred by conjugation in 95% (n = 71/75) of Efm and 97% (n = 28/29) of Efs, with a variable frequency (10^−1^–10^−8^). Transferable plasmids were identified as members of pRUM and Inc18 families or were mosaic plasmids of pRUM, Inc18 and pheromone plasmids (see sections below). Although some of these mosaic plasmids were detected in both Efm and Efs hosts, species-specific plasmid variants were predominant.

We have classified the enterococcal plasmids according to the content in rep/rel/TA systems, and RFLP profiles (Table 1, Figure 2). For the better interpretation of the results, we should keep in mind that members of the most common plasmid families classified in this and other studies as Inc18-like (pRE25, pIP501, pVEF1, pVEF2, pVEF3, pIP816, pEF1, pWZ909) or pRUM-like (pRUM, p5753cB, pS177) exhibit a high degree of *modular dissociability* or propensity for independent variation and shuffling, and may contain multiple replicons or be devoid of conjugation systems, thus making it very difficult to establish an accurate classification and to trace the origin of certain elements [9], [33]–[40]. See Clewell *et al.* for a comprehensive updated revision of enterococcal plasmids [9]. In the following sections we will describe vancomycin resistant plasmids of Efm and Efs.

**Table 1:** Plasmids identified in this study.

RFLP type	VanA modular profile	Size	No. isolates	Tn*1546*	PFGE type	City	Year
RFLP_1	Rep_17.2_::Rel_6_	60	1	A	ST190_H98	Coimbra	1998
RFLP_2	Rep_17.2_::Rel_6_	80	1	A	ST18_H92	Coimbra	2000
RFLP_8 c	Rep_17.2_::Rel_6_	120	1	PP2b	ST18_H70	Coimbra	2001
RFLP_9 c	Rep_17.2_::Rel_6_	60	1	PP2b	ST18_H70	Viseu	NI
RFLP_10 c	Rep_17.2_::Rel_6_	60	3	PP2b	ST18_H70, H93	Coimbra, Viseu	2002
RFLP_11	Rep_17.2_::Rel_6_	60	1	PP27	ST18_H87	Coimbra	2002
RFLP_3 b	Rep_17.2_::Rel_6_	95	3	PP4	ST18_H108	Coimbra	1998–2000-NI
RFLP_4 b	Rep_17.2_::Rel_6_	85	2	PP4	ST670_H90; ST18_H81	Coimbra	1997–2001
RFLP_7	Rep_17.2_::Rel_6_	50	1	PP5	NI	Viseu	2008
RFLP_6 b	Rep_17.2_::Rel_6_	80	1	PP5	ST18_H70	Coimbra	NI
RFLP_5 b	Rep_17.2_::Rel_6_	90	12	PP3, PP4, PP5, PP24	ST18_H78, H72, H94, H123, H129	Coimbra, Porto, Matosinhos	2001–2007
RFLP_5.2^b^	Rep_17.2_::Rel_6_	90	2	PP5	ST280_ H100	Porto, Viseu	2002–2003
RFLP_5.3^b^	Rep_17.2_::Rel_6_	90	1	PP5	ST125_H126	Matosinhos	2007
RFLP_5.3’^b^	Rep_17.2_::Rep_2_:: Rel_6_	85	2	PP5	ST132_H103	Coimbra	2002–2003
RFLP_5.2’^b^	Rep_17.2_::Rep_2_:: Rel_6_	90	1	PP4	ST132_H132	Coimbra	2001
RFLP_5.4’^b^	Rep_17.2_::Rep_2_:: Rel_6_	90	1	PP5	ST280_ H71	Viseu	2003
RFLP_5.5^b^	Rep_17.2_::Rep_2_:: Rel_6_	75	1	PP5	ST18_H125	Matosinhos	2007
RFLP_6.4	Rep_17.2_::Rep_2_:: Rel_6_	85	1	PP5	ST366_H99	Coimbra	2000
RFLP_20	Rep_17.2_::Rep_2_:: Rel_6_	30	1	A	ST656_H130	Vila Real	2008
RFLP_12 a	Rep_17.2_::Rep_2_:: Rel_6_	40	1	PP10	ST18_H83	Coimbra	1996
RFLP_13 b	Rep_17.2_:: Rel_6_::TA_Inc18_ a	60	1	PP5	ST280_ H71	Oporto	2002
RFLP_18	Rep_17.2_:: Rep_1_:: Rel_6_:: TA_Inc18_	50	1	PP13	ST132_H86	Oporto	2001
RFLP_16	Rep_17.2_:: Rep_1_:: Rep_2_:: Rel_6_::TA_Inc18_	50	1	PP13	ST18_H78	Oporto	2001
RFLP_17	Rep_17.2_:: Rep_1_:: Rep_2_:: Rel_6_::TA_Inc18_	110	1	PP13	ST132_H88	Oporto	2001
RFLP_19	Rep_17.2_:: Rep_1_:: Rep_2_:: Rel_6_::TA_Inc18_	60	3	PP13, PP20, PP23	ST132_H119	Oporto	2002
RFLP_19	Rep_17.2_:: Rep_1_:: Rep_2_:: Rel_6_::TA_Inc18_	60	2	PP17, PP20	ST368_SWH	Oporto	2001
RFLP_19.1	Rep_17.2_:: Rep_1_:: Rep_2_:: Rel_6_::TA_Inc18_	60	1	X	ST369_RP	Oporto	2003
RFLP_21	Rep_17.2_:: Rep_2_:: Rel_6_:: TA_pRUM_	65	1	PP15	ST719_H96	Oporto	2001
RFLP_22	Rep_17.2_:: Rep_2_:: Rel_6_:: TA_pRUM_	30	1	PP16	ST132_SWC	Oporto	2002
RFPL_27^d^	Rep_9_: Rep_2_: Rep_1_:: Rel_5_::TA_pAD1_	75–85	4	PP2a, PP4	ST6_HB, ST55_HG, ST159_HN	Coimbra	1996–2002
RFPL_27.3^d^	Rep_9_: Rep_2_: Rep_1_:: Rel_5_::TA_pAD1_	85	2	PP4	ST6_HB, ST159_HN	Viseu	2001–2002
RFPL_27.1^d^	Rep_9_: Rep_1_:: Rel_5_:: TA_pAD1_	75	1	PP4	ST6_HB	Oporto	2001
RFPL_27^d^	Rep_9_: Rep_2_: Rep_1_:: Rel_5_	85	1	PP9	ST132_H106	Coimbra	2000
RFPL_28.5	Rep_9_: Rep_2_	100	1	PP16	ST80_SWM	Oporto	2002
RFPL_28	Rep_9_: Rep_2_: Rel_5_:: TA_pAD1_	100	2	PP15, PP16	ST6_HB	Oporto	2001–2002
RFLP_29	Rep_9_: Rep_2_: Rel_5_	100	1	A	ST6_HB	Swine	2007
RFLP_30	Rep_9_: Rel_9_	100	1	A	ST30_HK	Oporto	2001
RFLP_24	Rep_2_	110	1	PP4	ST80_H80	Coimbra	1997
RFLP_14	Rep_2_	40	1	PP16	ST132_SWC	Oporto	2002
RFLP_15	Rep_1_:: Rep_2_	30	2	PP16	ST18_SWA, ST132_SWC	Oporto	2001
RFLP_23	Rel_6_	85	1	PP9	ST16_H74	Coimbra	2000
RFLP_26	Rep_20_:: Rel_6_	150	1	D	ST5_SA	Oporto	2002
RFLP_25	–	120	1	PP5	ST391_H124	Covilhã	2007

Abbreviations: RFLP, restriction fragment length polymorphism; ST, sequence type; NI, not identified.

a Plasmid type RFLP_12 (Rep_17.2/pRUM-like_ + Rep_2/pRE25/pEF1_ + Rel_6/pEF1_) contains a partial sequence of the replication gene of the RCR plasmid pEFNP1 (GenBank accession number AB038522), suggesting the integration of this RCR plasmid on the mobile element carrying Tn*1546* involving truncation of the rep_14/pRI1/pEFNP1_.

b Plasmid types RFLP_3, _4, _5, _6 and _13 (Rep_17.2/pRUM-like_ + Rel_6/pEF1_ and eventually containing Rep_1/pIP501_, Rep_2/pRE25/pEF1_ or TAI_nc18_) shared common bands and were identified in the same or different clonal backgrounds in different cities for extended periods of time.

c Plasmids types RFLP 8, _9 and _10 also shared a variable number of common bands.

d Plasmids showing patterns related to RFLP_27 (75–85 kb; rep_9/pAD1_ + rel_5/pAD1_ + rep_1/pIP501_ + *par*_pAD1_ and/or rep_2/pRE25/pEF1_) initially recovered from the widespread ST6-CC2 Efs clone in Coimbra in 1996 and other Efs (ST55 and ST159) and Efm clones contained similar IS*Ef1*-Tn*1546* variants (PP-2a, PP-4, PP-9). Other highly related mosaic Inc18-pAD1-related plasmids carrying IS*1216*-Tn*1546* were recovered from ST6 VREfs and ST80 VREfm isolates (type ˝II_Efs_ ˝, rep_9/pAD1_ +rel_5/pAD1_ + *par*_pAD1_ + rep_2/pRE25/pEF1_ *versus* type ˝II_Efm_ ˝, rep_9/pAD1_ + rep_2/pRE25/pEF1_).

### vanA plasmids of E. faecium

They were classified in two broad groups according to the plasmid replication modules and the background epidemiological context, i) pRUM-like variants (Rep_17.2/pRUM-like_+ Rel_6/pEF1_±Rep_1/pIP501_± Rep_2/pRE25/pEF1_/TA_Inc18_), ii) mosaics of Inc18-pRUM-like (Rep_2/pRE25/pEF1_ ± Rep_17.2/pRUM_/TA_Axe-Txe_). Highly transmissible pAD1-Inc18 mosaic plasmids from major Efs clones were also identified among Efm but they will be described in the next section.

**Figure 3:**
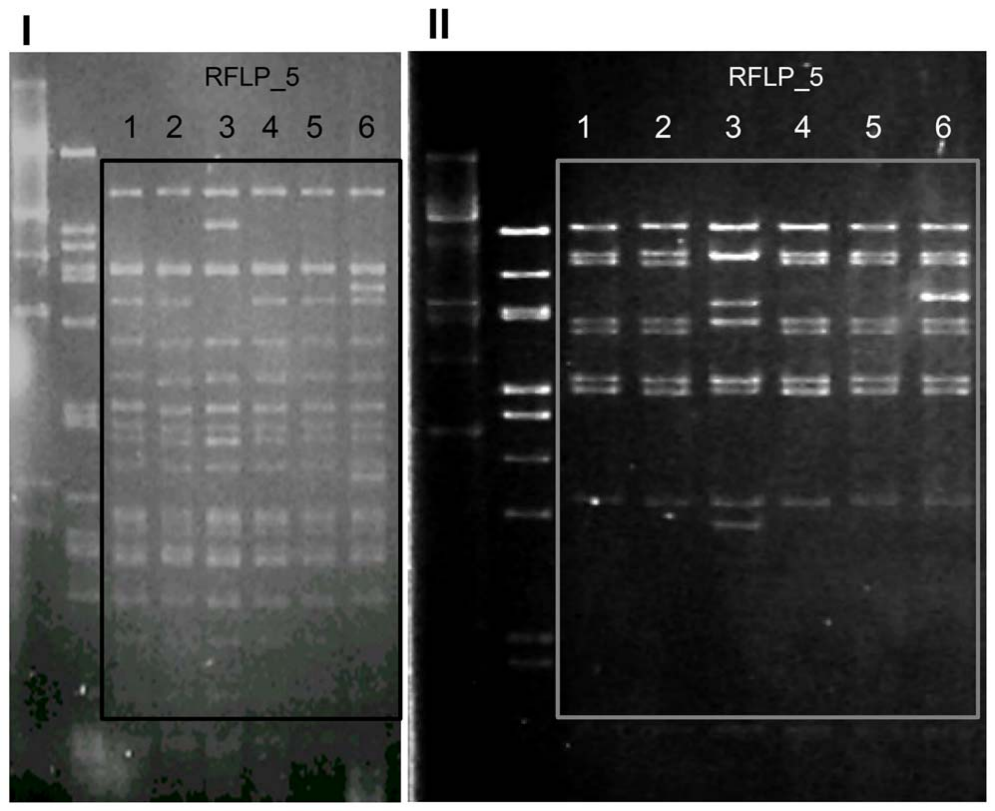
Restriction fragment length polymorphism patterns of plasmids showing RFLP_5 profiles after digestion with *Cla*I (I) and *Eco*RI (II) restriction enzymes (New England Biolabs Inc, UK). Lane 1, RFLP_5 (PFGE H78, ST18 Efm,); lane 2, RFLP_5 (PFGE H72, ST18 Efm); lane 3, RFLP 5.2 (PFGE H100, ST280 Efm), lane 4, RFLP 5 (PFGE H78, ST18 Efm); lane 5, RFLP_5 (PFGE H78, ST18 Efm); lane 6, RFLP_5.2’ (PFGE H132, ST132 Efm).

**Figure 4:**
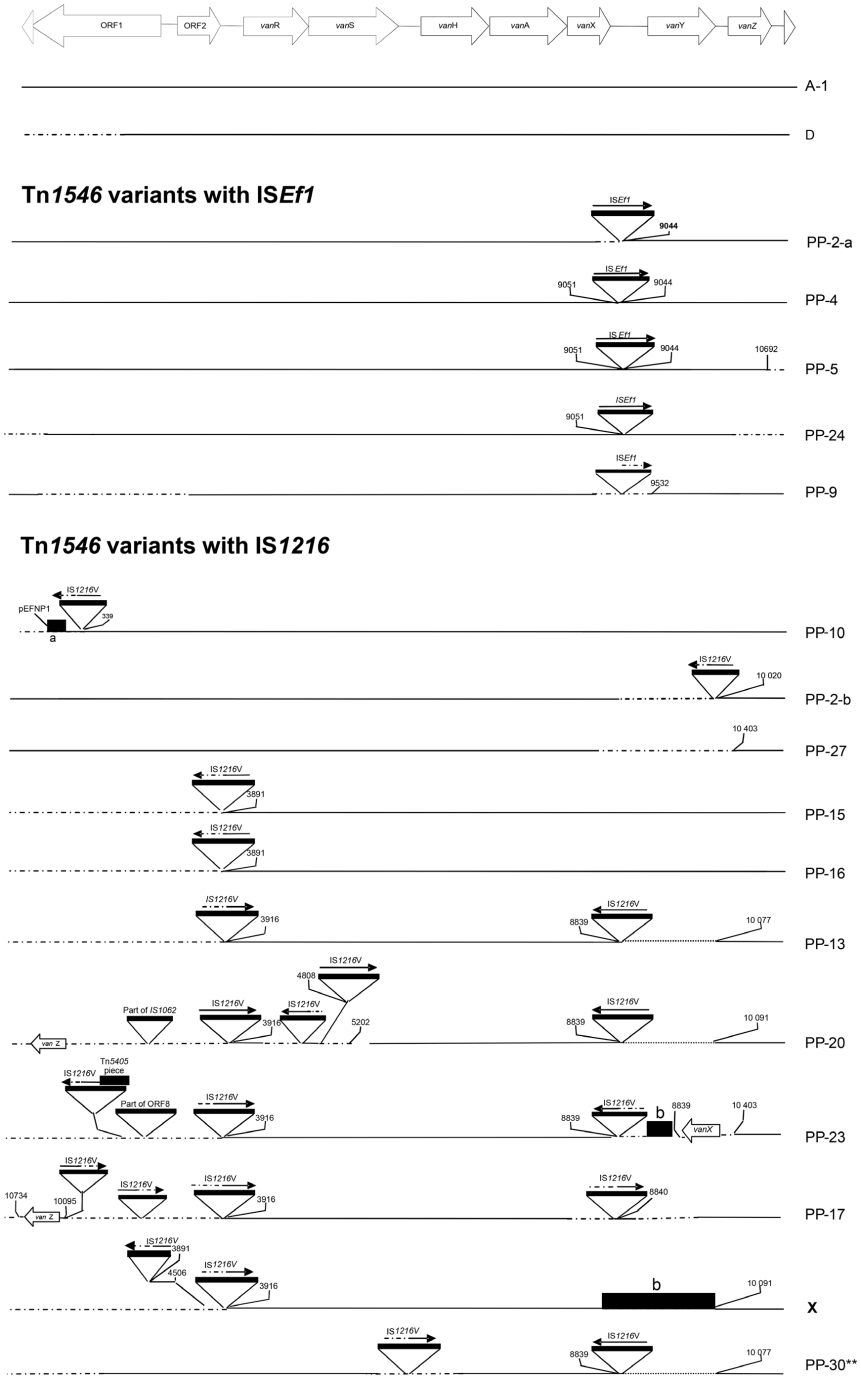
Genetic maps of Tn*1546* variants. Tn*1546* variants are represented as previously described by Novais et *al.* [27] although grouped differently and specific types have been further explored (PP10, PP30): Tn*1546* prototype A corresponds to the original sequence described by Arthur *et al*. [77] and D corresponds to Tn*1546* variants from animals. Tn*1546* variants with IS*Ef1* within *vanX-vanY* intergenic region (PP2a, PP4, PP5, PP9, PP24) and Tn*1546* variants with IS*1216* insertions at different positions (PP10, PP2b, PP13, PP15, PP16, PP17, PP20, PP23, PP27, PP30, X) are represented. The positions of genes and open reading frames and the direction of transcription are depicted with open arrows. IS elements are represented by triangles; other sequences are designated by rectangles. DNA insertions are represented highlighting the first nucleotide upstream and downstream from the insertion sites whenever known. Deletions are indicated by dots and discontinuous lines indicate sequences that were not characterized. (^a^) DNA sequence with homology to ORF3 (unknown protein product) and ORF1 (replication protein) of pEFNP1 plasmid (GenBank accession number AB038522). (^b^) DNA sequence with no match to any sequence available in GenBank. (*) PP23 was identified in an isolate susceptible to teicoplanin; this variant contained an insertion in the *vanY* gene that would affect the transcription of *vanZ* and it might explain the susceptibility to this glycopeptide as previously reported [27]. (**) PP30 was identified in an ST78 isolate susceptible to both glycopeptides (MIC against vancomycin and teicoplanin of 4 mg/L) carrying *vanA*-Tn*1546*. This variant contained alterations within the *vanS-vanH* intergenic region (an IS*1216* insertion), which is involved in the expression and regulation of the resistance to vancomycin, and it constitutes the first description of a *vanA* isolate phenotypically susceptible to vancomycin in Portugal.

- *pRUM derivatives* (rep_17.2/pRUM-like_+rel_6/pEF1_) of variable size (30–120 kb) were detected since the mid 90 s from a diversity of clonal backgrounds. pRUM plasmids showing different *Cla*I-digested DNA RFLP patterns were identified carrying a whole copy of Tn*1546* (RFLP_1, RFLP_2, RFLP_20, 30–80 kb), IS*1216::*Tn*1546* (RFLP_8–12, 40–120 kb) or IS*Ef1::*Tn*1546* (RFLP_3–7, RFLP_13, 50–95 kb). Despite the heterogeneity of plasmid profiles, RFLP_3–6 or RFLP_8–10 shared a variable number of common bands that suggest a relationship among them (see Table 1 and Figure 3 for details about relationships among plasmids). pRUM-like plasmids exhibiting distinct RFLP profiles and carrying different transposon variants were isolated in early and recent isolates of different clonal backgrounds (Figure 2). They include ST190, carrying a 60 kb plasmid RFLP_1 type; ST670 carrying a 85 kb exhibiting a RFLP_4 plasmid type; ST656 carrying a 30 kb plasmid designated as RFLP_20, and ST18, ST132, ST280, carrying different transposon variants. These results suggest multiple independent acquisitions of pRUM-like plasmids and further rearrangements with other elements, some plasmid variants being efficiently transferred among a diversity of different clones. It is of interest to highlight that epidemic ST18 PFGE types H83 (1996) and H92 (2000) harboured two pRUM-like plasmids. One was the rep_17.2/pRUM-like_::rel_6/pEF1_ vancomycin resistant plasmid showing RFLP_2 and RFLP_12 and the other was a 25 kb carrying a rep_17.1/pRUM_ gene and a copy of the Axe-Txe toxin-antitoxin system (rep_17.1/pRUM_+TA_Axe-Txe_) identical to the pRUM derivatives described to date (pRUM, p5753cB and pS177) (GenBank accession number GQ900487; Figure 2) [12], [38], [39] and other vancomycin resistant plasmids circulating at international level (Freitas *et al*., unpublished data). Diversification in the Rep sequences of these pRUM-like plasmids (homology of 96% at nucleotide level and 95% at protein level) might have resulted in the compatibility with similar (but not identical) plasmids in the same clonal background along extended periods of time.
- *Inc18 plasmids and mosaic Inc18-pRUM plasmids.* Clonally related ST132 and ST18 Efm isolates from Oporto contained Inc18 plasmids (Rep_1/pIP501_ ± Rep_2/pRE25/pEF1,_ RFLP_14–15) or mosaic plasmids of Inc18 and pRUM (Rep_2/pRE25/pEF1_+Rep_1/pIP501_+TA_Inc18_+Rep_17.2/pRUM-like_+Rel_pEF1_, RFLP_16–19), all carrying IS*1216*-Tn*1546* variants. Plasmids showing RFLP types 16–19 were highly similar (5 bands/12 bands in common), RFLP_19 being persistently recovered from clonally related ST132, ST368 and ST369 isolates, collected from hospitalized patients of HSA near by sewage plant and the river Douro from 2001 to 2003. This RFLP_19 has been also identified in a VREfm isolate recovered from swine in 2007 (Tn*1546* type “PP-31”, RFLP_19.1), highlighting the remarkable stability of particular VanA Inc18 plasmids in ensembles of related clones able to spread in different hosts [10]. A diversity of Tn*1546::*IS*1216* variants (PP-13, PP-17, PP-20, PP-23, PP-31 and X) which differed in the number of IS*1216* copies, the presence of insertions identified as short regions of Inc18-like plasmids or duplicated Tn*1546* sequence fragments in different orientations, were identified among related plasmids showing the RFLP_19 pattern (Table 1, Figure 4). These results illustrate the possibility of efficient intraclonal and intraplasmid diversification of Tn*1546::*IS*1216* variants. Acquisition of a *vanA*-Inc18 (rep_2/pRE25/pEF1_ + rep_1/pIP501_+ ω-ε-ζ) plasmid carrying a Tn*1546*::IS*1216* “variant”, predominant among poultry from Europe [41] by Portuguese strains containing VanA-pRUM (rep_17.2/pRUM-like_+rel_6/pEF1_) plasmids cannot be excluded. Recombination between pRUM::Tn*1546* and Inc18::Tn*1546* would explain duplicated Tn*1546* regions.
- *Megaplasmids*. Tn*1546* type “D” was located on a megaplasmid carrying rep_20/pLG1_ and rel_6/pEF1_ from isolates of a CC5 Efm clone spreading among swine and humans of different continents. This transposon has been previously associated with isolates from swine which frequently exhibit the G8234T mutation. The variable size (150–190 kb) of *vanA* megaplasmids linked to CC5 lineage has been previously reported [10].

### E. faecalis vanA plasmids

The *vanA* Efs plasmids were Inc18-pheromone-responsive mosaics, further classified in four main types on the basis of their RFLP patterns (RFLP_27–30), rep-rel/TA content, and replicase sequences. These plasmids have been documented in different Portuguese hospitals since the mid 90 s [26].

Plasmids showing highly related patterns designated as RFLP_27 (carrying IS*Ef1*-Tn*1546)* or RFLP_28 (carrying IS*1216*-Tn*1546*) were recovered from both Efs (ST6, ST55, ST159) and Efm (ST80, ST132). However, despite the similarity of their RFLP patterns, they differed in the rep/rel/TA content and transposon variant content (Table 1). Conversely, the finding of an ST117 Efs isolate from Oporto with two different *vanA* plasmids of 150 kb and 300 kb indicates acquisition and further recombination of widespread pRUM-*vanA* plasmids from Efm with narrow host pheromone responsive plasmids of Efs.

The observed differences in transposon variants and plasmid modules reflect frequent rearrangements during transfer of plasmids between Efs and/or Efm clonal backgrounds and also highlight the connectivity of these enterococcal populations resulting in the acquisition and generation of plasmids with enhanced host range.

### Fixation of *vanA*-Tn*1546* variants is associated with plasmid connectivity

Tn*1546* backbones were classified in three main groups corresponding to Tn*1546* with no insertion sequences (“type A” and “type D”) and variants containing IS*Ef1* (5 types) or IS*1216* (11 types) at different locations of the Tn*1546* backbone (Figure 4). Variants with a single copy of IS*Ef1* within the *vanX-vanY* region at nt 9044 were located on early (1996–1997) Efm plasmids identified as Inc18 and pRUM lacking Axe-Txe, and also on early (1996) Efs Inc18-pAD1 mosaics. Some of them were isolated from strains for more than one decade, which can be explained by their successful long-term recovered clonal and plasmid backgrounds.

Variants containing IS*1216* were mostly located on Inc18 plasmids or on mosaic plasmids Inc18-pRUM or Inc18-pAD1. Most variants contained the IS*1216* at 8839nt of the transposon (PP13, PP17, PP20, PP23, PP30) similarly to other Tn*1546* variants previously described in Europe [42]. Some of them also harboured different insertions corresponding to unknown sequences (X, PP23) or RCR plasmid sequences (PP10) [43] suggesting frequent recombination between acquired genes/plasmids and housekeeping Efm and Efs plasmids (Figure 4). Tn*1546* type D was specifically linked to megaplasmids from CC5 Efm from swine of different continents (Figure 2, Figure 4).

The presence of early plasmids carrying Tn*1546* belonging to different families suggests independent acquisitions of the transposon by pRUM and Inc18 plasmids, which would have been acquired by diverse Efm and Efs populations. Local fixation would be influenced by connectivity of plasmid and population backgrounds enabling further evolvability of transposon variants.

## Discussion

This paper shows the local dynamics of Tn*1546*-vanA among *Enterococci* is shaped by horizontal genetic transfer of pRUM and Inc18 plasmids and by recombination-driven evolution of them within and between Efs and Efm clones. The clonal diversity reported in this study has also been observed in areas where the spread of VRE has been documented [44]. Recent retrospective analysis of enterococcal populations suggests that the temporal evolution of the population biology of *Enterococci* is driven by a succession of epidemic waves of enterococcal human specific lineages, Efm ST78 and Efs ST6 emerging in the last decade at global scale similarly to that reported for other pathogens [19], [23], [24]. In Portugal, the population structure of VRE analysed in this study comprises isolates of main human Efm lineages, ST18 (ST18, ST132) being much more abundant than ST17 (represented by a single isolate of early ST16 lineage) [31], or ST78 (represented by sporadic ST80 and ST656, the first one linked to early VRE outbreaks) [25], [29]. It is worthwhile highlighting the recent detection of isolates of another Efm lineage in hospitals of the Oporto area (http://www.mlst.net) as ST117 Efm (ST78 lineage), which would reflect the increasing trend of isolates belonging to the ST78 lineage at international level. However, regional differences in the rates of VRE cannot be fully explained by clonal replacement dynamics since similar enterococcal clones appear widely distributed in areas with high and low rates of VRE (Tedim AP *et al.*, unpublished data). Instead, local conditions, including type and density of hosts, antibiotic usage, and transmission facilities, may influence regional differences in the proportions of VRE, as suggested by mathematical modelling studies on local trends of antibiotic resistance [45], [46]. Clones can locally evolve by variation, drift and short-distance migration, leading to changes in colonization ability, pathogenicity or even host range, the fittest clonal variants being able to facilitate the spread of antibiotic resistance [23], [47]–[50]. The observed clonal heterogeneity of the predominant ST18 lineage which comprises particular ST18 and ST132 strains widespread in different cities, highlights the role of certain efficiently transmissible clones in the dissemination of antibiotic resistance. Succesful clones can eventually be able to disseminate at international level as strains of ST6 Efs or ST280 Efm within main Efm human lineages driving or contributing the spread of different traits as Tn*1546* or Tn*1549* [51]. One remarkable fact is the similarity among PFGE patterns of isolates with different STs. Given the high content of plasmids and transposons of the isolates studied, and the frequent rearrangements identified among Efm and/or Efs isolates [21], chromosomal transfer can not be discarded. Recent phylogenomic analysis based on the degree of admixture among a diversity of isolates studied suggests that recombination is restricted to isolates within specific BAPS groups [19]. Most plasmids coding for vancomycin resistance are found in similar clonal backgrounds. This observation suggests that recombination does occur within isolates of similar BAPS groups as recently described [19]. However, the observed mosaicism and enhanced host range of particular plasmid variants indicates the existence of an unexpectedly high degree of connectivity between phylogenetically distant enterococcal populations and/or in bacterial genetic exchange communities integrating enterococci.

Broad host and narrow host plasmids carrying vancomycin resistance would have a high “*betweenness centrality”,* which is a pivotal index in network theory useful for measuring the load placed on the given node in the network as well as the node's importance to the network than just connectivity [52]. A recent *in silico* network analysis of all plasmid sequences available at the GenBank databases confirms very high ˝*betweenness* ˝ values for some Inc18 plasmids as pVEF3 (an Inc18 derivative highly spread among Efm from animals in Europe) [13], [37], and also for a pheromone-responsive plasmid pTEF1 (a plasmid recovered from ST6_Efs strain V583, highly related to the ST6 described in this work) [53] (unpublished data). Other plasmids with a high degree of *modular dissociability*, would be pRUM-like elements, which may enhance their complexity resulting in new configurations with enhanced *betweenness*. It is tempting to suggest that plasmid variability has contributed to intra-clonal diversification both in Efm and Efs, giving rise to a local wealth of clonal variants able to fully explore the local adaptive landscape. In fact, this and other studies demonstrate that selected variants of Inc18, pAD1, and pRUM plasmids can determine differences in the dynamics of VRE in different areas, further influencing the plasmid host range and the selection of specific clones within human adapted lineages. Examples of widespread plasmid variants of Inc18 or pRUM plasmids coding for vancomycin resistance have been reported recently. They included Inc18 widespread among Efm poultry isolates from Europe [13] or among Efs clinical isolates from the USA, the last one being able to transfer Tn*1546* to *S. aureus* [15]; and mosaics of pRUM variants containing Axe-Txe and Inc18 from humans in different continents (Freitas AR *et al*. unpublished data). The identification of chimeric pRUM-Inc18 plasmids containing rep/rel/TA of Inc18 sequences and Tn*1546* variants widely observed in poultry, hospitals and hospital sewage in the Oporto area reflects genetic exchanges between enterococci from different origins and highlights the need to enforce barriers to avoid the spread of multidrug resistance human pathogens to the environment and viceversa.

In this scenario, the genetic context of Tn*1546* seems to greatly influence the evolvability of the transposon and explains the high diversity of variants found in this and other studies [1], [27], [42], [54]. The frequent presence of insertions in the backbone of Tn*1546* and the abundance of IS*1216* and IS*Ef1* in enterococcal genomes [9], [55] makes homoplasic evolution of Tn*1546* in different backgrounds possible. However, other IS (IS*1251*, IS*1542*, IS*1476*, IS*19* and IS*1485*) linked to different plasmid and clonal backgrounds [9], [40] have been identified at different sites of Tn*1546,* thus suggesting that chance and selection are responsible to differences in variants collected in different areas. The widespread of Inc18 plasmids with a common origin in Europe [13], [56] indicates local fixation of Tn*1546* influenced by a founder effect and further connectivity of plasmid and population backgrounds enabling further evolvability of transposon variants as reported in this study.

Our results suggest that VRE spread is facilitated by selected clones of different lineages through strong interactive processes of clonalization and plasmid diversification that might occur at local scales. Despite the maintenance of significant gene flow, a sympatric, or more probably, parapatric bacterial clonalization process (when diverging populations share a common or neighbouring environment), might contribute to the formation of temporary genetic mosaics and the preservation of ecologically important genomic traits [57]. Such micro-evolutionary process will result in an array of clonal complexes forming a population structure able to exploit the local spatio-temporal patch heterogeneities [58]. Note that exploitation of connected microenvironments should accelerate evolution of antibiotic resistance [59]. The expected result of such a successful population structure is the local persistence of antibiotic resistant clones, and eventually the local fixation [60] of vancomycin-resistance [46].

In summary, this study highlights the relevance of studying the local microecology of genes, elements, lineages and populations to decipher the robustness of the trans-hierarchical networks connecting these evolutionary elements in order to describe and predict the local evolvability of vancomycin-resistance [61]. Traditional surveillance studies are *one-off cross* sectional surveys focused on single traits as epidemic strains, genes or mobile genetic elements over limited periods of time which only gives *one shot* view that precludes addressing the long-term dynamics of antibiotic resistance. The more comprehensive approach described in this study is needed for understanding in depth the evolution of complexity in multihierarchical systems as those involved in the spread of antibiotic resistance among the populations of bacterial human pathogens.

## Materials and Methods

### Bacterial strains and epidemiological background

One hundred four VRE clinical isolates carrying Tn*1546* from different regions of Portugal, 75 VREfm and 29 VREfs, were analyzed in this study. They included: i) clinical isolates from hospitals of Coimbra (Hospital Universitário de Coimbra, HUC), Oporto (Hospital Santo António, HSA), Viseu (Hospital de São Teotónio, HST); Matosinhos (Hospital Pedro Hispano, HPH), Vila Real (Hospital S. Pedro, HVR) and Covilhã (Centro Hospitalar da Cova da Beira, CHCB) located in Northern and Central Portugal (62 Efm and 26 Efs; 1996–2008); ii) isolates from waste waters of hospitals (HSA and Hospital de São João, HSJ) (10 Efm and 3 Efs), and iii) isolates from the estuary of the River Douro (3 Efm) recovered in the Oporto area during 2001–2003. Part of the isolates analyzed in this work corresponds to strains from previous surveillance studies [25]–[27], [62]; this paper constitutes the first description of isolates obtained during 2007 and 2008. Contemporary Portuguese VRE isolates of animal origin were used for comparative analysis of lateral transfer events [10].

Susceptibility against 15 antibiotics was determined by the agar dilution method following CLSI standard guidelines. Clonal relatedness was established by pulsed-field gel electrophoresis (PFGE), banding patterns were interpreted according to criteria previously suggested for long-term studies, and multilocus sequence typing (MLST) as described elsewhere (http://efaecium.mlst.net) [25], [63]–[65].

The presence of putative virulence traits [collagen-binding adhesin (*acm*), enterococcal surface protein (*esp*), hyaluronidase (*hyl_E. faecium_*), cytolysin/hemolysin (*cyl*), gelatinase (*gelE*) and aggregation substance (*agg*)] was searched by using PCR as described [66], [67].

### Genetic context of Tn*1546*

Characterization of Tn*1546* backbone was determined by amplification of overlapping transposon fragments and further sequencing of PCR products [27], [68]. We have accomplished the analysis for the isolates not studied in previous surveys and have interpreted the resulting transposon diversity (this study) [27], under the light of the plasmid and clonal backgrounds identified in this geographical area.

### Plasmid analysis

Isolates (n = 62 Efm and n = 13 Efs) representing the clonal diversity observed in both species were selected for plasmid characterization (Table 1, Figure 2). The content and size of plasmids from transconjugants obtained by filter mating were determined by using either the technique described by Barton *et al.* (plasmids >10 kb) or the alkaline lysis extraction method of Kado & Liu (plasmids <10 kb) [51], [69], [70]. Classification of *E. faecium* plasmids was based on the presence of specific modules for replication (rep-initiator proteins), mobilization (relaxases) and stability (toxin-antitoxin systems). *Relaxases* (rel) were sought by a multiplex-PCR-based relaxase typing method which differentiates relaxases of the MOBQ, MOBP, MOBC and MOBV families related to 27 known plasmids [9], [71] (Francia MV, unpublished data). *Replication initiator proteins* (rep) were investigated by amplification of 24 replicons, which allows discriminating among DNA sequences from more than 100 published Gram-positive plasmids [9], [72]. Designation of rep sequences pointed out the plasmid type in which they were initially identified, as well as the numeric nomenclature originally used by Jensen *et al*. (Figure 2 ´s footnote) [72]. *Toxin-antitoxin systems* (TA) previously identified among streptococci and enterococci (Axe-Txe, ω-ε-ζ *par, maz*EF) or Gram-negative bacteria (*rel*BE) were detected by PCR [73]. PCR products were sequenced in order to confirm the specificity of the method and to analyze similarities with other well-characterized plasmids. Genomic location of the Tn*1546* and the rel/rep/TA sequences was determined by hybridization of *vanA* and *rel/rep/TA* specific probes obtained by PCR from DNA from reference plasmids with S1 or I-*Ceu*I digested genomic DNA from representative strains [51], [69]. Structural relationship between plasmids of similar size was established by comparison of their RFLP patterns obtained after digestion with different restriction enzymes (*Eco*RI, *Hind*III and *Cla*I; see Figure 3). Plasmid DNA was obtained by using a modified protocol based on the alkaline lysis method described by Handwerger *et al.* [74] consisting of increasing two-fold the volume of lysozyme, SDS/NaOH and acetate potassium solutions, extending the incubation period in potassium acetate solution for at least three hours, precipitating the supernatant obtained after extraction with phenol-chloroform using ethanol-acetate potassium solution (2∶0.1 vol/vol) at 25°C for at least 2 hours, and resuspending final DNA pellets in 30 µl of water for further enzyme digestion analysis.

### Molecular techniques

Southern blot DNA transfer and hybridization were performed by standard procedures [75]. The *vanA* and rep/rel/TA/bac probes used in the hybridization assays were generated by PCR using well known positive controls as template DNA. Labelling and detection were carried out using Gene Images Alkphos Direct Labelling system kit, following the manufacturer's instructions (Amersham GB/GE Healthcare Life Sciences UK Limited). PFGE was performed as described previously [76] using the following conditions: switch time of 5 s to 25 s for 6 h, followed by 30 s to 45 s for 18 h (S1 nuclease); 5 s to 30 s for 22 h, 14°C, and 6 V/cm^2^ (I-*Ceu*I) and 1 s to 20 s for 26 h, 14°C, and 6 V/cm^2^ (*Sma*I).

### Plasmid sequences

Analysis of nucleotide and aminoacid sequences revealed two types of sequences amplified with primers used for identification of rep_17/pRUM._ They were 100% (designated as Rep_17.1/pRUM_) or 97% (96% identity at amino acid level; designated as Rep_17.2/pRUM-like_) homologous to that of RepA_pRUM (GenBank accession number AF507977). Most Rep_1/pIP501_ aminoacid sequences were 98%–100% identical to RepE_pIP816, a member of the Inc18 family (GenBank accession number AM932524), and to a lesser degree to pRE25, pTEF1 or pSM19035; and Rep_2/pRE25/pEF1_ showed 96%–100% amino acid identity to that of pEF1 (GenBank acc. no. DQ198088). Sequences identified as Rel_6/pEF1_ showed 98%–100% homology to orf34_pEF1. Relaxases of the *E. faecalis* pheromone-responsive plasmids identified in this study displayed a high homology with those of known enterococcal pheromone plasmids pAD1, pAM373 and pTEF1 (orf57_pAD1, GenBank acc. no. AAL59457; EFA0025_pTEF1, GenBank AE016833; and EP0019_pAM373, GenBank acc. no. NC_002630). That of plasmid showing RFLP_27 showed a 67–84% homology with the above mentioned pheromone enterococcal plasmids but 94% identity with a MobC relaxase (annotated as a hypothetical protein) from a vancomycin-resistant *S. aureus* strain (GenBank acc. no. EIK35827).
